# Sustained improvements in EQ-5D utility scores and self-rated health status in patients with ankylosing spondylitis after spa treatment including low-dose radon – an analysis of prospective radon indication registry data

**DOI:** 10.1186/s12891-022-05691-1

**Published:** 2022-08-03

**Authors:** Antje van der Zee-Neuen, Victoria Strobl, Heidemarie Dobias, Julia Fuchs, Johannes Untner, Wolfgang Foisner, Martina Knapp, Sebastian Edtinger, Martin Offenbächer, Markus Ritter, Bertram Hölzl, Martin Gaisberger

**Affiliations:** 1 Center for Physiology, Pathophysiology and Biophysics, Institute for Physiology and Pathophysiology, Salzburg, Austria; 2 Gastein Research Institute, Paracelsus Medical University, Strubergasse 22, 5020 Salzburg, Austria; 3 Institute of Nursing Science and Practice, Paracelsus Medical University, Salzburg, Austria; 4 Center for Public Health and Healthcare Research, Paracelsus Medical University, Salzburg, Austria; 5 Ludwig Boltzmann Institute for Arthritis and Rehabilitation, Salzburg, Austria; 6 Gastein Healing Gallery, Bad Gastein, Austria; 7 Cure and Rehabilitation Center, Bad Hofgastein, Austria; 8 Stiftung Kurtherme Badehospiz, Bad Gastein, Austria; 9 Baerenhof Health Care & Rehabilitation Center, Bad Gastein, Austria; 10 Department of Physical Medicine and Rehabilitation, Kardinal Schwarzenberg Klinikum, Schwarzach Im Pongau, Austria; 11 Center for Physiology, Pathophysiology and Biophysics, Institute for Physiology, Pathophysiology and Biophysics, Nuremberg, Germany; 12 Kathmandu University School of Medical Sciences, Dhulikhel, Nepal; 13 Department of Internal Medicine, Landesklinik St. Veit Im Pongau, SALK, Paracelsus Medical University, Salzburg, Austria

**Keywords:** Ankylosing spondylitis, Quality of life, Spa therapy, Radon

## Abstract

**Background:**

Patients with ankylosing spondylitis (AS) have significantly lower quality of life (QoL) than the general population. Holistic interventions addressing QoL comprise spa- or balneotherapy including radon. These interventions have shown to be beneficial in reducing pain and improving QoL in AS-patients. We explored the association of spa-therapy including low-dose radon with QoL in AS-patients over an extended time period.

**Methods:**

Registry data collected for the “*Radon indication registry*” in the Austrian Gastein valley comprising data on QoL (EuroQol EQ-5D) directly before the treatment (baseline), directly(t1), 3 (t2); 6(t3) and 9(t4) months after the treatment, age, sex and body mass index (BMI) were analysed. Linear regression models explored the association of measurement time with 1) EQ-5D-5L utilities and 2) EuroQol visual analogue scale (VAS) score. Alterations of 0.05 (utilities) and 5.00 (VAS) were considered clinically relevant.

**Results:**

Two-hundred-ninety-one AS-patients were included in the analyses. Forty-four percent (*n* = 128) were women, the mean age was 52 (SD 10) and the average BMI was 26 (SD 4). Utilities (t1: 0.09 [0.07;0.11]; t2: 0.08 [0.06; 0.10]; t3: 0.06 [0.05;0.09]; t4: 0.04 [0.02;0.06]) and VAS (t1: 11.68 [9.38; 13.97]; t2: 12.20 [9.78; 14.61]; t3: 9.70 [7.24; 12.17]; t4: 6.11 [3.57; 8.65]) were significantly higher at all timepoints compared to baseline. Improvements were clinically relevant at all timepoints in case of the VAS and until 6 months after treatment for the utilities.

**Conclusion:**

AS-patients who received spa therapy including radon show significantly and clinically relevant improvements in Qol until 6–9 months after treatment.

## Introduction

Ankylosing spondylitis (AS) is the most common form of the rheumatic disease group of spondyloarthritis. It occurs in approximately 23.8 per 10,000 Europeans [1] and is more prevalent in men than women. AS affects the axial skeleton leading to inflammatory back pain, damage to physical structures as well as impairments in physical functioning. These impairments may result in reduced participation and decreased quality of life (QoL). [1–4] The growing understanding of QoL as key factor when measuring the effectiveness of healthcare interventions as well as the embracement of bio-psychosocial models rather than just biological models for the evaluation of health emphasize the relevance of interventions focussing on the improvement of QoL [5, 6].

Previous research has pointed out that AS-patients have significantly lower QoL than the general population but that pharmacological treatment is beneficial in improving their QoL. Particularly the combination of anti-TNF-α therapy in combination with physical exercise may reduce the adverse effect of AS on QoL [7, 8].

However, the evidence on the effectiveness of alternative or complementary non-pharmacological interventions in improving QoL in AS patients is still limited. Common symptom-oriented interventions like physiotherapeutic treatment are effective in the reduction of disease activity and pain as well as the improvement of functional capacity [9] Yet, holistic interventions have the potential of addressing a wider range of the AS-patient’s health state including mental health and participation in daily life [10, 11]. Those aspects are particularly relevant when assessing QoL from a patient’s perspective.

Holistic interventions for AS regularly comprise multidisciplinary treatments including spa- or balneotherapy/speleotherapy. Still, little is known about the effect of these interventions on QoL. Kamioka et al. summarized the body of knowledge in an overview of systematic reviews with meta-analysis based on randomized controlled trials of balneotherapy and spa-therapy from 2000 to 2019 and did not identify any review focussing on spa-therapy in relation to QoL [12]. A limited number of studies specifically addressed the effectiveness of combined spa‐exercise therapy on QoL. For example, Colina et al. demonstrated that in AS patients, combining pharmacological treatment with spa-therapy resulted in significantly better QoL than pharmacological treatment alone six months after treatment initiation [13]. A randomized controlled trial by van Tubergen et al. showed that QoL, expressed by EuroQol-5D utilities, was significantly higher in patients that received spa-therapy (one with and one without radon treatment) compared to those who received usual care until 40 weeks after the treatment. In this study, the application of utilities enabled valuation of QoL from a societal perspective (i.e., utility values accounted for preferences the society has for a particular health state).[14].

Among spa-therapies, treatment with low-dose radon has shown to be effective in achieving long-term pain reduction in persons with musculoskeletal diseases (including AS) [11, 15–19] and showed promising results with regard to improvements in functionality [20, 21] as well as in QoL [22, 23]. However, to the best of our knowledge, until now no data exist on the association of spa therapy including radon with systematically monitored QoL in patients with AS over an extended period of time while accounting for both, QoL from a societal perspective and individually perceived QoL.

Therefore, the aim of the current study was to explore whether spa treatment including low-dose radon results in sustained significant and clinically relevant improvement of QoL in patients with AS.

## Methods

The current study concerns a longitudinal analysis of prospectively collected registry data from the ongoing “Radon indication registry for the assessment of pain reduction, increase of quality of life and improvement in body functionality throughout low-dose radon hyperthermia therapy” (registration ID ISRCTN67336967; https://doi.org/10.1186/ISRCTN67336967) in the valley of Gastein in Austria. The registry collects data from individuals visiting the valley of Gastein for the purpose of spa-treatment including radon for a variety of rheumatic diseases. They are recruited for participation by the physicians of participating spa centres in the Gastein valley. Data are collected following informed consent by means of standardized paper questionnaires that are completed by participants directly before commencement of the treatment (baseline), directly after the treatment and 3; 6 and 9 months after the treatment. Questionnaires are sent out by medical employees of the participating centres for the last three timepoints and are handed over in person for the first two timepoints. Questionnaires are then sent back to the spa centres and handed over to the Gastein research institute after pseudonymization. There a research assistant enters the data manually into the database.

### Population

**Fig. 1:**
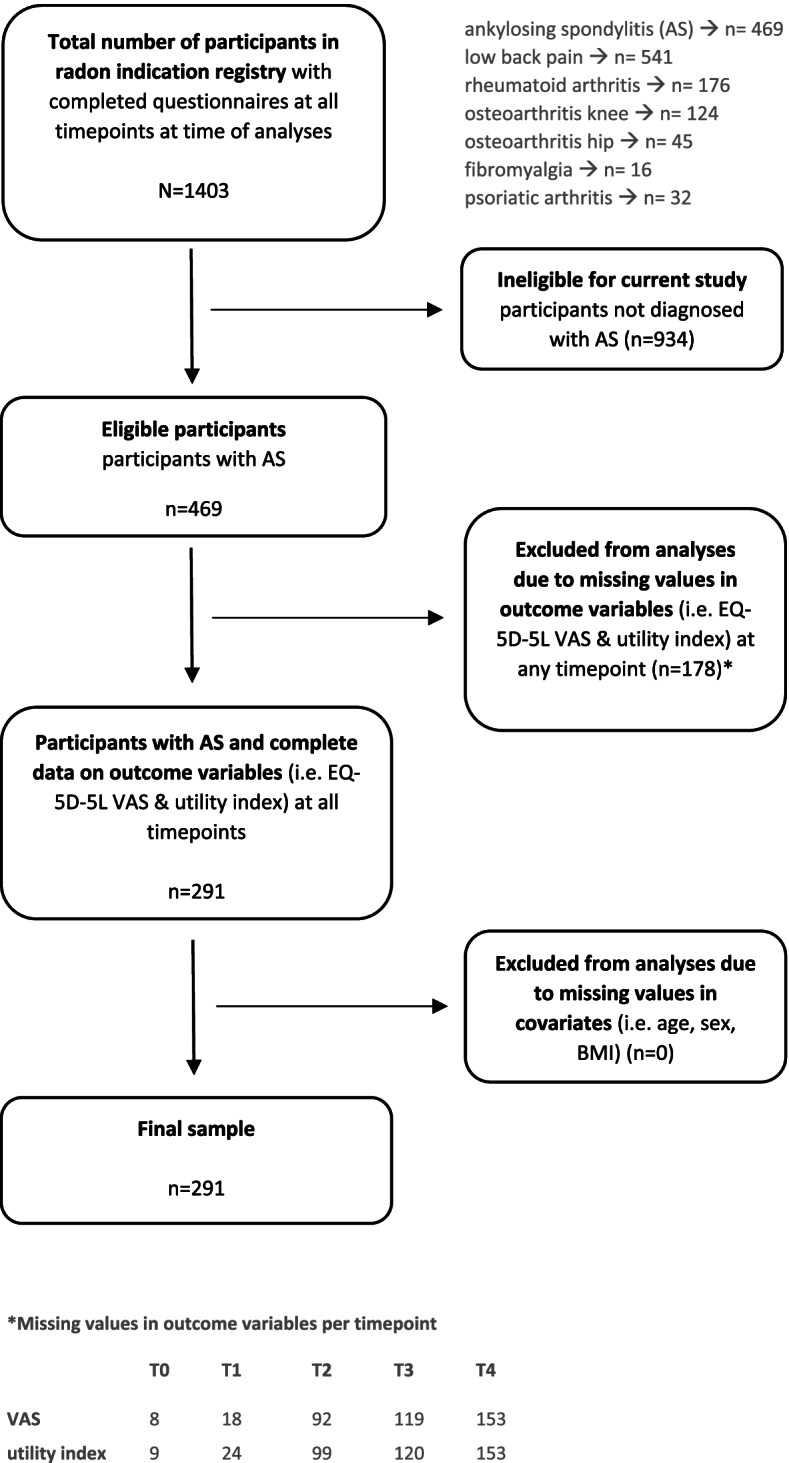
Flow chart of study sample selection

At the time of analysis for the current manuscript, the radon indication registry comprised 469 AS patients, 541 patients with low back pain, 176 patients with rheumatoid arthritis, 124 patients with osteoarthritis (OA) of the knee, 45 patients with OA of the hip, 16 patients with fibromyalgia and 32 patients with Psoriatic arthritis. For the current study, only AS patients were included if they had provided complete data at each timepoint for all variables included in the analyses. (Fig. 1).

### Intervention

The intervention consisted of an individualized (i.e. based on local spa-physicians prescription) spa-treatment including radon in the valley of Gastein in the Austrian Alps with an average duration of 17.5 days (SD 3.5). This so-called low-dose radon balneo/speleo therapy (LDRnBST; radon-therapy) is part of a holistic treatment program for patients with AS and is applied in terms of balneo- and/or speleotherapy. The former includes bathing in water (~ 37 °C) with low activity of radon (average 707.57 (SD 233.27)Bq/l measured by liquid scintillation, Triathler™ LSC Hidex) as applied by the local facilities according to standardized treatment regimens. The thermal water consists of several mineral substances e.g., sodium – 80,01 mg/l; potassium – 5,71 mg/l; calcium – 19,84 mg/l; magnesium – 0,75 mg/l; hydrogen carbonate – 57,91 mg/l; chloride – 24,96 mg/l; flourid – 5,61 mg/l; sulphate – 130,67 mg/l; carbon dioxide – 6,87 mg/l.[24].

A balneotherapy intervention including low-dose radon consists of approximately 10 baths with a duration of 20 min. Speleotherapy including low-dose radon describes the process of relaxation while being exposed to a low activity of radon (average 44 kBq/m^3^, as indicated by healing gallery), high humidity (70–100%) and mild hyperthermia (37–41.5 °C) in the healing gallery of Gastein (a former gold mine located in moderate altitude (1270 m) above sea level) for an average time of 60 min on alternate days (i.e. an average of 11 speleotherapy sessions).[25] Both forms of radon therapy evoke a mild form of hyperthermia and increase the body temperature.

### Outcomes

The EuroQol EQ-5D-5L (*© EuroQol Research Foundation. EQ-5D™, hereafter referred to as EQ-5D)* is a self-reported questionnaire consisting of two parts, a descriptive system comprising 5 dimensions of health (i.e., mobility, self-care, usual activities, pain/discomfort, anxiety/depression) and a visual analogue scale (VAS) capturing participant’s self-rated health status on a 0–100 scale with higher values representing better health. Using the unique score from each of the 5 dimensions of health a utility index score can be calculated (i.e., von Neumann-Morgenstern utility value for current health) [26].

Single values for each of the 5 dimensions reflect the level of problem with each dimension resulting in an individual health state. This health state can be converted into a weighted health state by applying scores from the EQ-5D preference weights extracted from the general population which can take a value from 0 (death) to 1 (full health).

The EQ-5D utility index and EuroQol VAS were used as outcome variables for the current study. In absence of Austrian population weights, German population weights were used to calculate the EQ-5D utility index [27].

### Main independent variable of interest and covariates

The timepoint of survey completion by the participants was used as main independent variable of interest. Covariates were chosen a priori and included age (in years), sex (men/women) and body mass index (BMI; BMI = weight[kg]/height[m]^2^) due to their already established influence on health and health related QoL [28–30].

### Statistical analyses

First, descriptive statistics were used to characterize the sample in terms of age, gender and BMI at baseline (i.e., directly before the intervention) and to describe the EQ-5D utility index and VAS-score for each of the timepoints of measurement. Next, two linear regression models were computed to explore the association of timepoint of measurement with a) the EQ-5D utility index and b) the EuroQol VAS-score while adjusting for age, sex and BMI. After each model, margins and their 95% confidence interval (CI) were calculated to produce specific age, gender and BMI standardized estimates for the utility index and VAS score.

*P*-values ≤ 0.05 were considered statistically significant. A change of ≥ 0.05 in the EQ-5D utility index and of ≥ 5.00 in the EuroQol VAS was considered clinically relevant [31, 32].

## Results

**Fig. 2:**
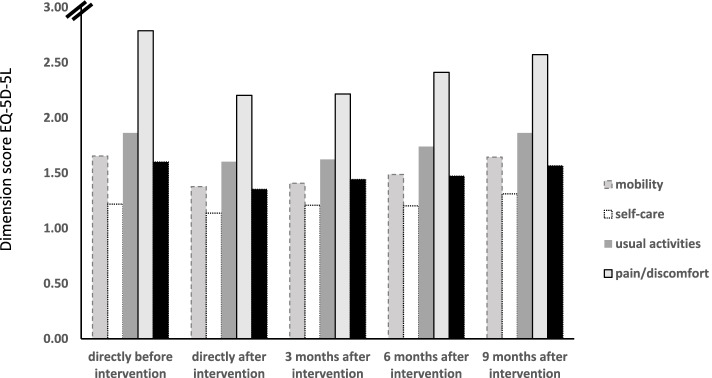
Unadjusted average course of health based on EQ-5D health dimensions (dimension score range 1–5 with lower scores representing better health)

The final sample included in the analyses consisted of 291 participants who provided complete data for all timepoints (Fig. 1). The sample consisted of 128 women, the mean age was 52 years and the average BMI was 26. Table 1 shows the unstandardized EQ-5D utility index and VAS scores for each timepoint. Figure 2 illustrates the course of the dimensions (i.e. mobility, self-care, usual activities, pain/discomfort, anxiety/depression) based on which the utility index was calculated.

**Table 1:** Characteristics of study population at baseline and directly, 3, 6 & 9 months after spa-treatment including radon

	Baseline^a^	Time after intervention			
		directly	3 months	6 months	9 months
Age, mean (SD); range	51.80 (10.14); 19.00–79.00				
Women, n (%)	128 (44.00)				
Body mass index, mean (SD); range	26.37 (4.25); 18.37–39.56				
EQ-5D utility index^1^, mean (SD); range	0.79 (0.17); 0.01–1.00	0.88 (0.11); 0.36–1.00	0.87 (0.12); 0.24–1.00	0.85 (0.13); 0.19–1.00	0.83 (0.16); 0.08–1.00
EQ-VAS^2^, mean (SD); range	62.51 (18.01); 15.00–100.00	74.88 (17.10); 2.00–100.00	74.76 (15.41) 20.00–100.00	71.46 (15.52) 18.00–98.00	67.23 (18.00) 20.00–100.00
Clinically relevant improvement EQ-5D utility index^3^, n (%)		164 (56.36)	160 (54.98)	130 (44.67)	107 (36.77)
Clinically relevant improvement EQ-VAS^3^, n (%)		207 (70.10)	191 (65.64)	170 (58.42)	139 (47.77)

^a^directly before intervention

^1^von Neumann-Morgenstern utility value for current health based on 5 dimensions of EuroQol (i.e. mobility, self-care, usual activities, pain/discomfort, and anxiety/depression), range 0–1 (i.e. 0 = death, 1 = perfect health)

^2^self-rated health status on a graduated (0–100) scale, 0 = ‘The worst health you can imagine’, 100 = ‘The best health you can imagine’

^3^clinically relevant improvement. ≥ 0.05 for EQ-5D utility index and ≥ 5.00 for EQ-VAS)

### Health over time EQ-5D utility index

The age, sex and BMI standardized association of timepoint of measurement and utility index showed that at each timepoint, the index value was significantly higher than the baseline value indicating better health. Directly, 3 months and 6 months after the intervention the index value increased by 0.09 [95%CI 0.07;0.11], 0.08 [95% CI 0.06; 0.10] and 0.06 [95% CI 0.05;0.09], respectively. Since all increases exceeded 0.05, they reflected a clinically relevant change [31].

Nine months after the intervention the utility index was still increased (0.04 [95% CI 0.02;0.06]) but this improvement was not clinically relevant. (Table 2).

**Table 2:** Adjusted association of timepoint of measurement with EQ-5D utility index and VAS^1^

	EQ-5D utility index B [95% CI]	EQ-VAS B [95% CI]
Timepoint (reference = directly before treatment)		
Directly after spa-treatment	0.09 [0.07;0.11] *^1^	11.68 [9.38; 13.97] *^1^
3 months after treatment	0.08 [0.06; 0.10] *^1^	12.20 [9.78; 14.61] *^1^
6 months after treatment	0.06 [0.05;0.09] *^1^	9.70 [7.24; 12.17] *^1^
9 months after treatment	0.04 [0.02;0.06] *	6.11 [3.57; 8.65] *^1^
Age, mean (SD); range	-0.001 [-0.001;0.000]	-0.18 [-0.26; -0.11] *
Sex (reference = women)	0.01 [-0.00;0.03]	2.21 [0.56; 3.87] *
Body Mass Index	-0.002 [-0.004;-0.001] *	-0.22 [-0.43; -0.04] *

Associations are calculated by means of multivariable linear regression

^1^number of participants included = 291

^*^significant at *p* ≤ 0.05

^1^clinically relevant improvement (i.e. ≥ 0.05 in the EQ-5D utility index and of ≥ 5.00 in the EQ-VAS)

### Health over time euroQol VAS

The age, sex and BMI standardized association of timepoint of measurement and EuroQol VAS showed that at each timepoint, the VAS score was significantly higher than the baseline value indicating better health. Directly, 3 months, 6 months and 9 months after the intervention the VAS score increased by 11.68 [95%CI 9.38; 13.97], 12.20 [95% CI 9.78; 14.61], 9.70 [95% CI 7.24; 12.17] and 6.11 [95% CI 3.57; 8.65], respectively. These increases reflected a clinically relevant change since they were larger than 5.00 [32]. (Table 2).

**Fig. 3:**
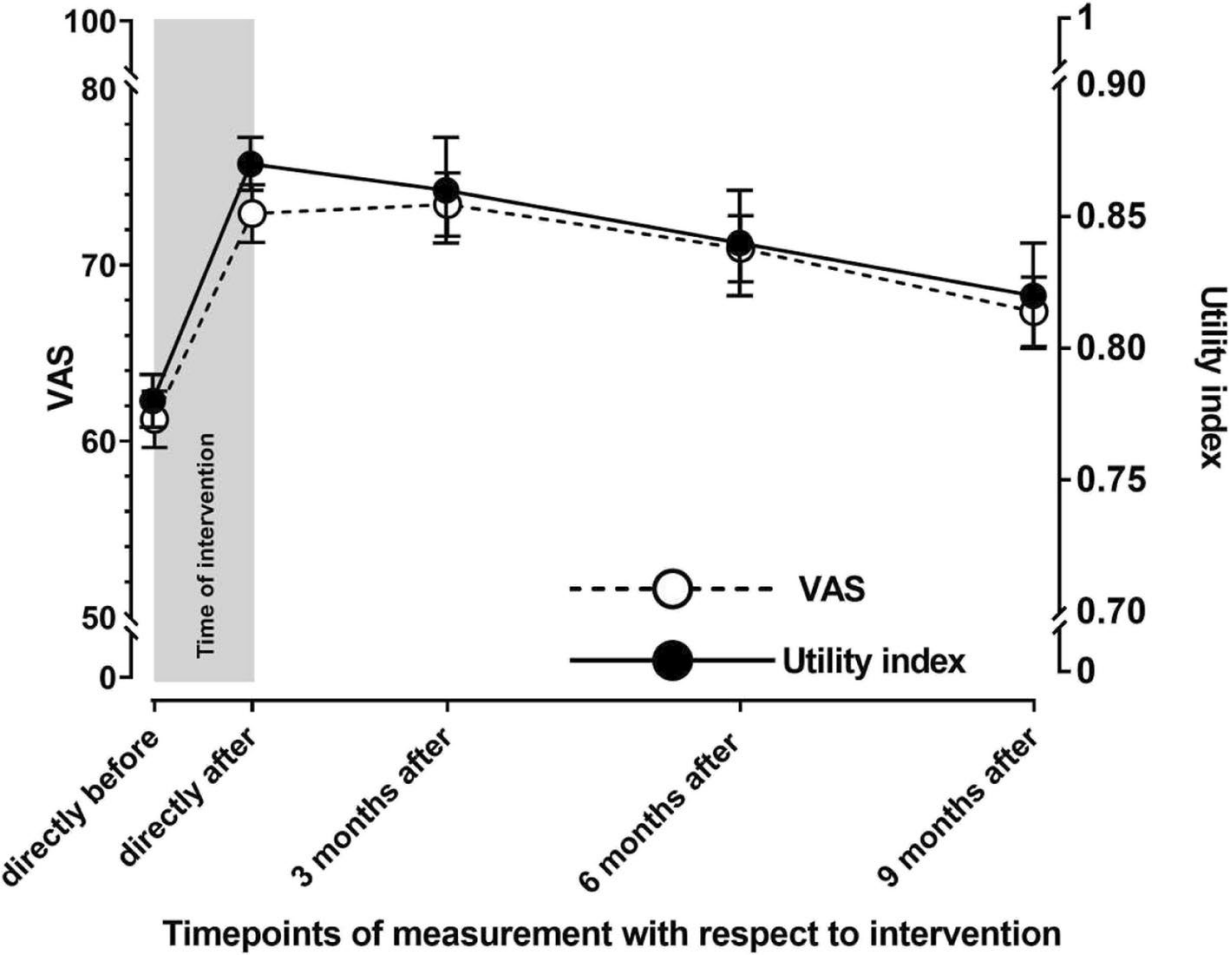
Age, sex and BMI adjusted course of self-reported health state based on EuroQol VAS scores and utility index score (utility index range 0–1; VAS-score range 0–100 with higher scores representing better health)

Figure 3 illustrates the age, sex and BMI adjusted course of self-reported health state based on EuroQol VAS scores and utility index score.

## Discussion

To our knowledge, this is the first time that systematically collected registry data have been used to explore the association between spa-therapy including radon with alterations in QoL in AS patients over a period of 9 months. Significant improvements in QoL were seen immediately and were sustained until 9 months after the intervention. Predicted mean scores based on linear regression models showed these improvements were clinically relevant until 6 months after the intervention in case of the EQ-5D utility index and until 9 months after the intervention in case of the EQ-VAS while accounting for individual differences in patient characteristics. Generally, these findings are in agreement with other studies focussing on the benefits of spa therapy for QoL in AS patients.[13, 14, 22, 23].

Some differences were found in the course of the utility index compared to the course of the VAS-score. The utility index showed the largest improvement directly after the intervention while the VAS score was highest 3 months after the intervention. The latter is in agreement with other publications focussing on symptom relief and alterations in QoL through spa-therapy including radon which show a delayed therapy response. For example, van Tubergen et al. found the same delay when focussing on the EQ-5D utility index [14]. However, in our study the delay was only observed in the VAS-score. A possible explanation might be found in the different population preferences accounted for in the calculation of utilities in the current study (German preference weights) compared to the study of van Tubergen et al. (Dutch preference weights). Yet, the unadjusted illustration of the 5 dimensions of health prior to the application of preference weights shows the same course of improvement suggesting that another explanation is more likely. Selection bias might be one: The observation may be attributable to the specific population included. Provision of data for the radon registry is voluntarily and participants included in the current study had provided complete data at all timepoints. This might indicate high motivation attributable to favourable treatment effects that are more precisely represented by the utility index than by the VAS score. An interesting side finding was that men had significantly higher EQ-VAS scores compared to women independent of their age, BMI or the timepoint of measurement. Previous research showed, that women with AS have less improvement in AS related outcome measures compared to men. However, the reason for this phenomenon remained unclear [33]. In the current study the difference between men and women was not clinically relevant and only occurred in case of the VAS but not the utility index, which might suggest that perception of health plays a relevant role.

Clinically relevant changes attributable to spa therapy including radon have, to our knowledge, not been addressed by previous studies. The current evidence points out that in our cohort of AS-patients clinically relevant improvements in QoL can be sustained until 6 months (utility index) or even 9 months (VAS) after intervention. From a clinical perspective, this indicates the benefits of a repetitive treatment pattern. To achieve stable results, a periodic intervention should be scheduled every 6 months.

### Limitations and Strengths

As in all studies based upon registry data limitations arise from the fact that data collection is not monitored or performed by the researcher and that data on confounders is somewhat limited [34].

In the current study data on the frequency of interventions prior to the first timepoint of measurement were not systematically collected. This might have resulted in biased baseline values as participants who have received the intervention repeatedly likely have a better baseline health state than those who receive the intervention for the first time leading to a potential underestimation of the improvement in first-time participants. Moreover, it should be considered that in pre-post estimations, the nature of self-reported measures may lead to biased responses due to the direct effect of an intervention on participant’s perception of health rather than their actual health (i.e. response shift) [35].

Confounders in the association of QoL with AS have been identified in previous literature and might have affected the current analyses as well. For example, a lower level of education and being a smoker is associated with lower QoL but this information was not available.[36].

Strengths of the study include a relatively large study sample with complete data over an extended period of time as well as the independence of data collection. Since data on the effectiveness of spa therapy including radon on the improvement of QoL in AS patients is still scarce, the current study provides relevant insights and opportunities for further research among other patient populations, and in comparison with usual care.

## Conclusion

In conclusion, the current study reveals that AS patients who received spa-therapy including low-dose radon show significantly and clinically relevant improvements in Qol and that these improvements are sustained for up to 9 months. It may be considered a valuable (complementary) treatment option for this purpose. Extrapolation of the results may support the decision of policy makers and insurances to refund bi-annual spa therapy including radon for patients with AS.

## Data Availability

The datasets used and/or analysed during the current study are publicly available from the open data storage platform Zenodo using the following link: https://doi.org/10.5281/zenodo.5926209.

## Abbreviations

AS: Ankylosing spondylitis
BMI: Body mass index
CI: Confidence interval
EQ: EuroQol
QoL: Quality of life
RnIR: Radon indication registry for the assessment of pain reduction, increase of quality of life and improvement in body functionality throughout low-dose radon hyperthermia therapy
VAS: Visual analogue scale
